# Young and healthy C57BL/6 J mice performing sprint interval training reveal gender- and site-specific changes to the cortical bone

**DOI:** 10.1038/s41598-018-19547-z

**Published:** 2018-01-24

**Authors:** Robin Hollinski, Anja Osterberg, Stefan Polei, Tobias Lindner, Daniel Cantré, Thomas Mittlmeier, Brigitte Vollmar, Sven Bruhn, Brigitte Müller-Hilke

**Affiliations:** 1 Institute for Immunology, Rostock University Medical Center, Rostock, Germany; 2 Core Facility Multimodal Small Animal Imaging, Rostock University Medical Center, Rostock, Germany; 3 Institute of Diagnostic and Interventional Radiology, Rostock University Medical Center, Rostock, Germany; 4 Department for Trauma, Hand and Reconstructive Surgery, Rostock University Medical Center, Rostock, Germany; 5 Institute for Experimental Surgery, Rostock University Medical Center, Rostock, Germany; 6 Department of Exercise Science, Rostock University, Rostock, Germany

## Abstract

Physical exercise is considered to impede the bone loss associated with physiological ageing however, a training program that efficiently leads to bone accrual in the healthy does not yet exist. We turned to the C57BL/6 J mouse and designed a sprint interval training for treadmill that was tailored to the individual performance limits. It consisted of four weeks with five training sessions each, followed by another four weeks with three. After completion of the training period, mice were sacrificed and the hind legs were analyzed via µCT and MRI for changes in bone parameters and muscle volume, respectively. Increased performance limits in both sexes confirmed an effect of the treadmill training. However, while male tibiae after eight weeks revealed a significant reduction of cortical bone mass at the distal metaphysis, the cross sectional analysis of female tibiae showed a transient decrease of cortical bone mass after four weeks that was reversed into a significant accrual after eight weeks of training and occurred over the entire length of the tibia. The observed net reduction of female bone mass after four weeks of training is suggestive of a remodelling process which may be delayed in the males.

## Introduction

Bone is a dynamic organ that is constantly being remodeled depending on both, systemic mediators and site-specific reactions after physical impact. The former implies hormonal changes that are of relevance during puberty as well as later in life when diminishing levels of sex hormones are associated with osteopenia and osteoporosis^1,2^. The latter, physical impact, has intensely been investigated and training programs for humans revealed that high-impact or odd-impact loading like hurdling, karate, volleyball or soccer and racquet games lead to the greatest bone accrual^3,4^. On the other hand, low impact sports like swimming, cycling and long distance running failed to associate with an increase in bone mass^4^.

The greatest limitation of the exercise trials performed with humans are the lack of longitudinal studies, varying compliance of exercise among participants, diverse genetic backgrounds, the bias of professional athletes vs. non-trained individuals and most importantly, the restriction of bone imaging which implies DEXA or pQCT at best. Exercise programs performed with animals are therefore a logical consequence and have numerously been performed and reviewed^5^. In summary, *in vivo* mechanical loading turned out to yield the best reproducibility of results and suggested that the most important parameters to consider when designing a setting for bone accrual are frequency, strength and duration of the strain as well as recovery in between strains^6,7^.

In order to design a physiological training program that in the future can be applied to prevent osteopenia in patients at risk, we previously turned to the STR/ort mouse as a model and the treadmill as training device^8^. As an improvement of geometric and mechanical bone properties following moderate treadmill training in young and middle-aged mice had already been described^9,10^, the aim of our previous study was to combine existing training programs with the insight gained from *in vivo* mechanical loading experiments. We thus designed a high intensity sprint interval training (SIT) with the sprints providing a high frequency of strains and the intervals reducing the fatigue adoption of bone as a reaction to an otherwise monotonous stimulus. Surprisingly, in our previous experiments, both sexes failed to improve geometric alongside mechanical bone properties^8^. In order to find out whether these findings were strain specific, we here turned to the C57BL/6 mouse and hypothesized that the low bone mass phenotype of C57BL/6 mice might be more permissive for bone accrual following physical strain. Based on previous observations that i) treadmill training in the rat affected the cortical more than the cancellous bone^11,12^ and that ii) the distal tibia supports greater weight per bone area^13^, we expected the changes to the bone – independent of whether they would be bone accrual or bone loss – to occur in the cortical bone of the distal tibia.

## Results

### Training increased maximum running speeds

**Figure 1:**
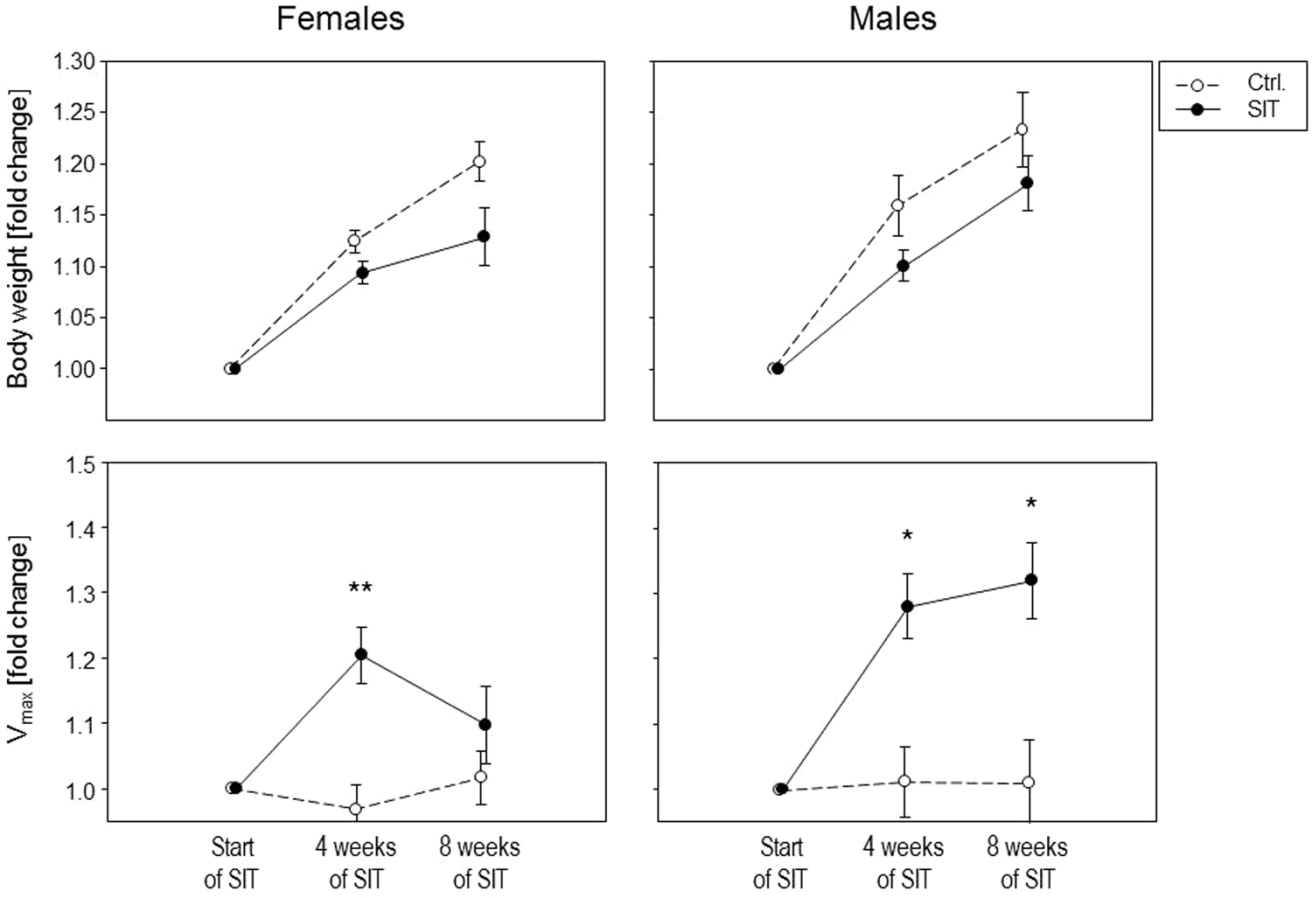
Trained mice developed physiologically and increased their individual performance limits. The increases in weight (upper panels) and maximum running speeds Vmax (lower panels) are presented for females and males after four and eight weeks of training, respectively. The data points correspond to normalized means ± SEM for the controls (open symbols) and the mice that underwent SIT (filled symbols). Note that the values at four weeks of training are pooled from mice that were sacrificed immediately after the second R-T-E and those that went on for another four weeks of training. Mann-Whitney tests were performed for comparisons; *P < 0.05; **P < 0.01.

In order to assess the effect of the treadmill training, mice were regularly weighed to monitor their physiological development and were also tested for improved running speeds. Figure 1 shows that controls (N = 5 males and N = 12 females) and trained mice (four weeks of SIT: N = 6 females, eight weeks of SIT: N = 5 males and N = 6 females) developed comparably and this held true for males and females alike. Of note, the controls gained more weight than the trained mice however, this difference did at no time point reach statistical significance (Fig. 1, upper panels). Moreover, males weighed significantly more than age-matched females of the same group and the P-values describing this difference ranged between 1.34 × 10^−5^ and 1.76 × 10^−3^.

Importantly, the maximum running speeds of trained mice significantly increased after four weeks of high frequency SIT in both females (P = 0.0026) and males (P = 0.0193) (Fig. 1, lower panels). After additional four weeks of reduced frequency SIT, the males still showed a significantly increased maximum velocity compared to the non-trained controls (P = 0.0362). However, four weeks of reduced frequency SIT in the females led to reduced running speeds and no more significant differences compared to the control group. Peak and active recovery velocities during the SIT were 24–31.8 m/min and 12–16.2 m/min, respectively for the females and 24.6–30.6 m/min and 12–15 m/min, respectively for the males.

### Sprint interval training in the males led to a significant reduction of cortical bone mass at the distal tibiae

We hypothesised that our extremely strenuous SIT would exert a sufficiently high strain for bone accrual at the hind legs. After completion of eight weeks of SIT we therefore subjected the femora and tibiae to micro computed tomography (µCT). The analysis of trabecular bone was restricted to the distal femoral and proximal tibial metaphyses and revealed no SIT related changes at all (Table 1). Likewise, there were no changes to the cortical bone at femoral and tibial mid-diaphyses (Table 1).

**Table 1:** Male bone parameters following 8 weeks of Sprint Intervall Training (SIT).

Group	Trabecular*					Cortical*				
	BV/TV [%]	Tb.Th. [mm]	Tb.N. [1/mm]	SMI	BMD [g/cm³]	B.Ar/ T.Ar[%]	B.Ar [mm²]	T.Ar [mm²]	Cs.Th [mm]	BMD [g/cm³]
**Femora**										
Ctrl.	7.827 ± 0.897	0.051 ± 0.002	1.539 ± 0.147	2.452 ± 0.091	0.198 ± 0.007	51.876 ± 0.426	1.050 ± 0.031	2.025 ± 0.063	0.220 ± 0.004	1.178 ± 0.010
SIT	10.079 ± 2.219	0.049 ± 0.004	1.989 ± 0.340	2.315 ± 0.143	0.206 ± 0.012	52.153 ± 0.626	0.961 ± 0.045	1.842 ± 0.088	0.209 ± 0.006	1.166 ± 0.006
P-value	n.s.	n.s.	n.s.	n.s.	n.s.	n.s.	n.s.	n.s.	n.s.	n.s.
**Tibiae**										
Ctrl.	12.734 ± 2.287	0.055 ± 0.003	2.258 ± 0.295	2.213 ± 0.110	0.210 ± 0.016	64.712 ± 0.781	0.974 ± 0.030	1.504 ± 0.033	0.221 ± 0.006	1.085 ± 0.009
SIT	11.614 ± 2.198	0.053 ± 0.003	2.129 ± 0.292	2.340 ± 0.122	0.200 ± 0.017	63.218 ± 0.660	0.903 ± 0.030	1.429 ± 0.045	0.212 ± 0.003	1.070 ± 0.007
P-value	n.s.	n.s.	n.s.	n.s.	n.s.	n.s.	n.s.	n.s.	n.s.	n.s.

*Trabecular parameters were evaluated at distal metaphyses for the femora and at proximal metaphyses for the tibiae. Cortical parameters were evaluated at femoral and tibial diaphysis.

Data represent mean ± SEM (n = 5). Kruskal-Wallis tests (with post tests) were performed for comparisons.

**Figure 2:**
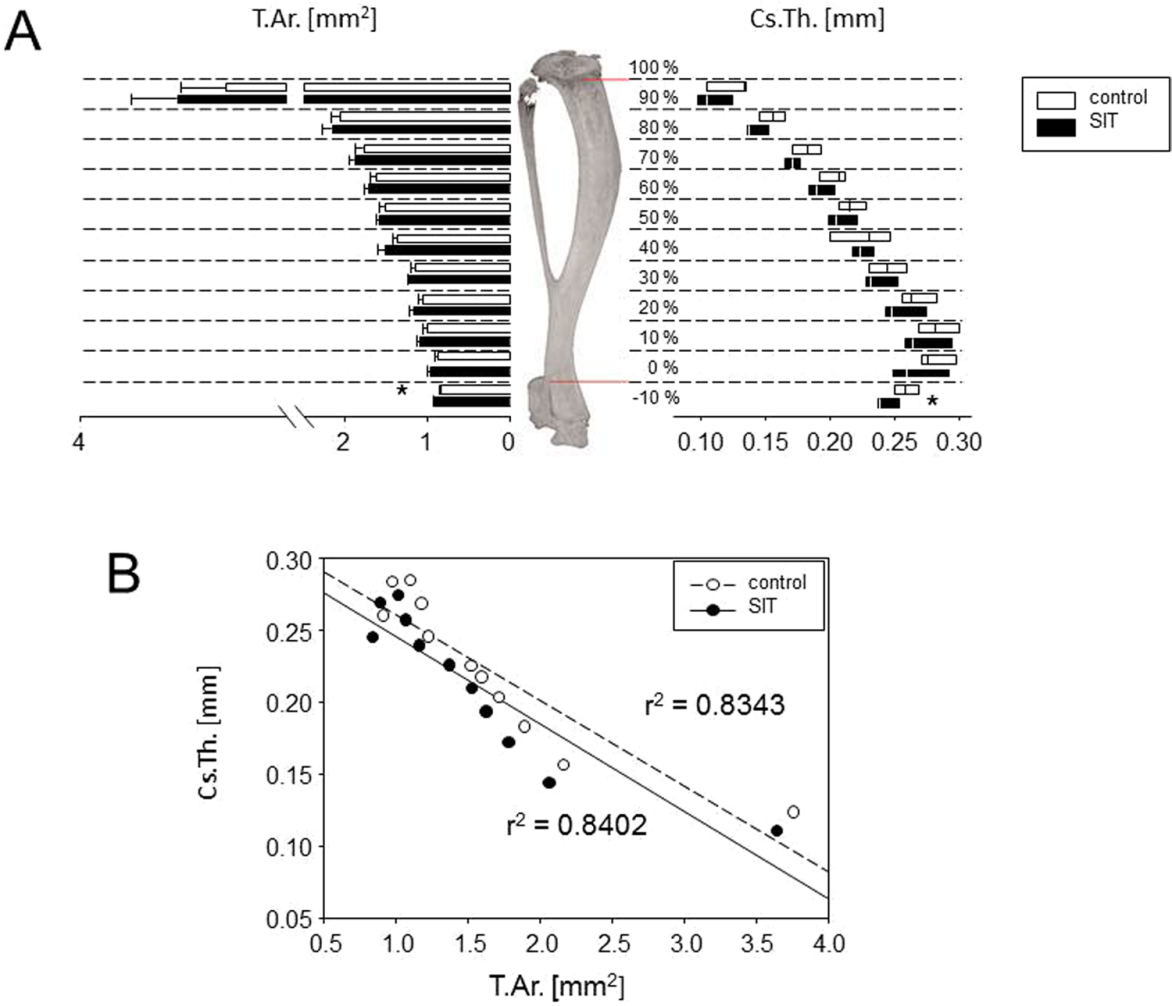
Sprint interval training in the males led to a significant reduction of cortical bone mass at the distal tibiae. (**A**) Bar and box plots compare the means of tissue areas (T.Ar.) at the various tibial positions as well as the corresponding medians of cross-sectional thicknesses (Cs.Th.). Comparisons are shown for trained vs control males. Data following Gaussian distribution are presented as means and bars and were compared performing Student’s t-tests. Data not following Gaussian distribution are presented as box-plots and were compared performing Mann-Whitney tests. Resulting p-values <0.05 are indicated by *. (**B**) Plot shows significant negative correlations between the cross-sectional thicknesses (Cs.Th.) and the tissue areas (T.Ar.) of trained males and non-trained male controls. Correlation coefficients (r) were calculated according to Pearson.

However, as we expected the tibiae to be most responsive to physical strain, we analysed these bones more closely. In detail, the tibiae were divided into eleven intervals of identical length, 10 between the upper and lower reference limits and one below. Contrary to our expectations, eight weeks of SIT led to significant reductions of T.Ar and Cs.Th. at the distal metaphyses and thus indicated periosteal bone loss (see Fig. 2A). In order to further address whether these exercise-induced changes to the cortical bone were dictated by the geometric shape, we correlated the cross-sectional thickness (Cs.Th.) to the tissue area (T.Ar.). Figure 2B illustrates negative correlations for trained and control mice with comparable correlation coefficients (r^2^ = 0.8402 and 0.8342, respectively) and corresponding P-values smaller than 0.0001. Our results thus suggest that independent of the exercise, the tibial Cs.Th. is highest in regions of low T.Ar. and this is where exercise-induced bone loss occurs in the males.

### Female femora showed no response to the Sprint Interval Training

Female mice were subjected to either four or eight weeks of SIT and the subsequent µCT analysis of their hind legs allowed for a cross sectional evaluation of bone parameters. We defined purely age related changes as changes observed between the controls at four and at eight weeks. In contrast, SIT related changes were defined as changes observed between the trained mice and their age matched controls at each time point (four and eight weeks of training, respectively). Likewise, SIT related changes also included those observed between trained mice that had undergone four and eight weeks of SIT, as long as the corresponding age-related changes were less significant.

**Figure 3:**
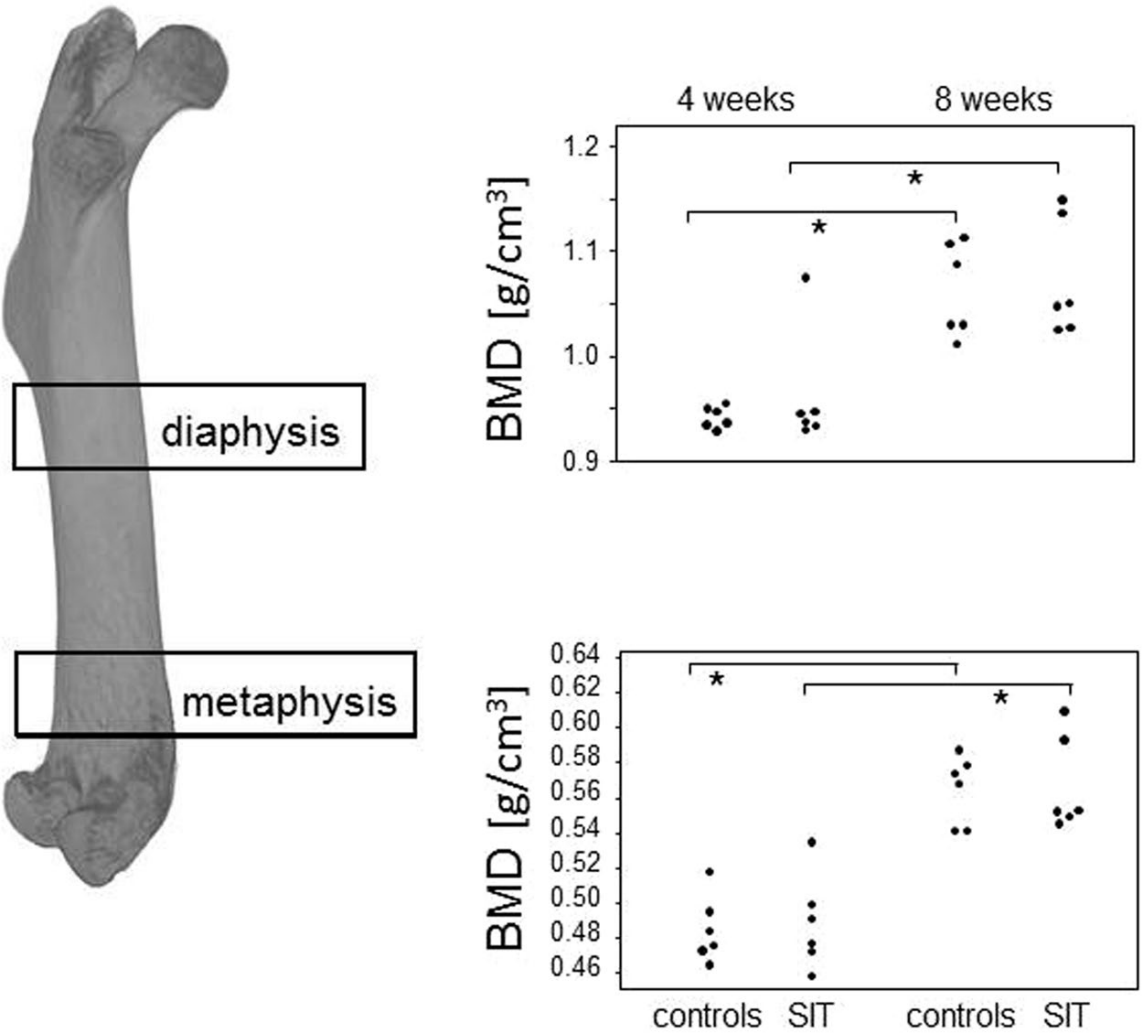
Female femora showed no response to the Sprint Interval Training but revealed age related increases in BMD. (A) femoral regions analysed via µCT were the mid-diaphysis for cortical and the distal metaphysis for trabecular bone parameters. Dot plots show the corresponding comparisons for BMD (cortical BMD at diaphysis in the upper panel and trabecular BMD at distal metaphysis in the lower panel). Significant differences resulting from Kruskal-Wallis with post tests are marked by asterisks, *P < 0.05.

For the femora, trabecular and cortical bone parameters were assessed at the distal metaphysis and at the diaphysis, respectively. The resulting mean values are presented in Table 2 and show that there were significant changes to the BMD for both, cortical and trabecular bone. However, these changes were merely age related, as the differences between the controls at four and eight weeks were as significant as the changes observed between trained mice after four and eight weeks of SIT. Importantly, there were no further differences between the trained and control mice, neither after four nor eight weeks of SIT and this held true for trabecular and cortical parameters alike (Table 2 and Fig. 3). In summary, the female femora showed no response to the SIT.

**Table 2:** Female bone parameters following 4 and 8 weeks of Sprint Intervall Training (SIT).

Group	Trabecular*					Cortical*				
	BV/TV [%]	Tb.Th. [mm]	Tb.N. [1/mm]	SMI	BMD [g/cm³]	B.Ar/T.Ar[%]	B.Ar [mm²]	T.Ar [mm²]	Cs.Th [mm]	BMD [g/cm³]
**Femora**										
4 weeks of training										
Ctrl.	2.844 ± 0,328	0.051 ± 0.002	0.559 ± 0.059	2.831 ± 0.035	0.485 ± 0.008	50.968 ± 0.694	1.069 ± 0.020	2.099 ± 0.056	0.222 ± 0.002	0.942 ± 0.004
SIT	1.978 ± 0.607	0.050 ± 0.005	0.371 ± 0.100	2.998 ± 0.064	0.489 ± 0.011	50.112 ± 0.743	0.974 ± 0.052	1.939 ± 0.084	0.210 ± 0.007	0.961 ± 0.023
8 weeks of training										
Ctrl.	3.133 ± 0.749	0.051 ± 0.005	0.577 ± 0.092	2.856 ± 0.045	0.565 ± 0.008	51.981 ± 0.719	1.004 ± 0.068	1.926 ± 0.107	0.219 ± 0.009	1.063 ± 0.018
SIT	3.370 ± 0.994	0.047 ± 0.005	0.643 ± 0.131	2.783 ± 0.048	0.567 ± 0.011	51.724 ± 0.475	1.043 ± 0.057	2.014 ± 0.094	0.221 ± 0.008	1.072 ± 0.023
P-value	n.s.	n.s.	n.s.	n.s.	0.0006^a^	n.s.	n.s.	n.s.	n.s.	0.0027^a^
**Tibiae**										
4 weeks of training										
Ctrl.	6.908 ± 0.710	0.074 ± 0.002	0.931 ± 0.078	2.651 ± 0.045	0.093 ± 0.004	63.174 ± 0.493	0.948 ± 0.022	1.501 ± 0.040	0.221 ± 0.001	0.881 ± 0.010
SIT	6.212 ± 0.568	0.073 ± 0.001	0.853 ± 0.070	2.624 ± 0.075	0.086 ± 0.004	61.314 ± 0.297	0.903 ± 0.024	1.473 ± 0.037	0.208 ± 0.004	0.858 ± 0.010
8 weeks of training										
Ctrl.	4.229 ± 0.415	0.070 ± 0.002	0.602 ± 0.055	2.732 ± 0.063	0.086 ± 0.003	64.104 ± 0.504	0.995 ± 0.013	1.552 ± 0.016	0.221 ± 0.003	0.906 ± 0.009
SIT	5.401 ± 0.880	0.073 ± 0.003	0.725 ± 0.094	2.603 ± 0.043	0.092 ± 0.006	62.930 ± 0.324	1.023 ± 0.009	1.626 ± 0.017	0.229 ± 0.002	0.914 ± 0.009
P-value	n.s.	n.s.	0.0319^b^	n.s.	n.s.	n.s.	0.0037^c^	0.0095^c^	0.0018^c^	0.0078^c^

*Trabecular parameters were evaluated at distal metaphyses of femora and at proximal metaphyses of tibiae. Cortical parameters were evaluated at femoral and tibial diaphyses. Data represent mean ± SEM (n = 4–5). Kruskal-Wallis tests (with post tests) were performed for cross sectional comparisons. ^a^The significance results from the comparison of the controls at the four and eight weeks time points and of the trained mice after 4 and 8 weeks of SIT. ^b^The significance results from the comparison of the controls at the four and the eight weeks time points, only. ^c^The significance results from the comparison of the trained mice after four and eight weeks of SIT, only.

### Sprint interval training related changes to the cortical bone of female tibiae occurred at both, endosteal and periosteal surfaces

**Figure 4:**
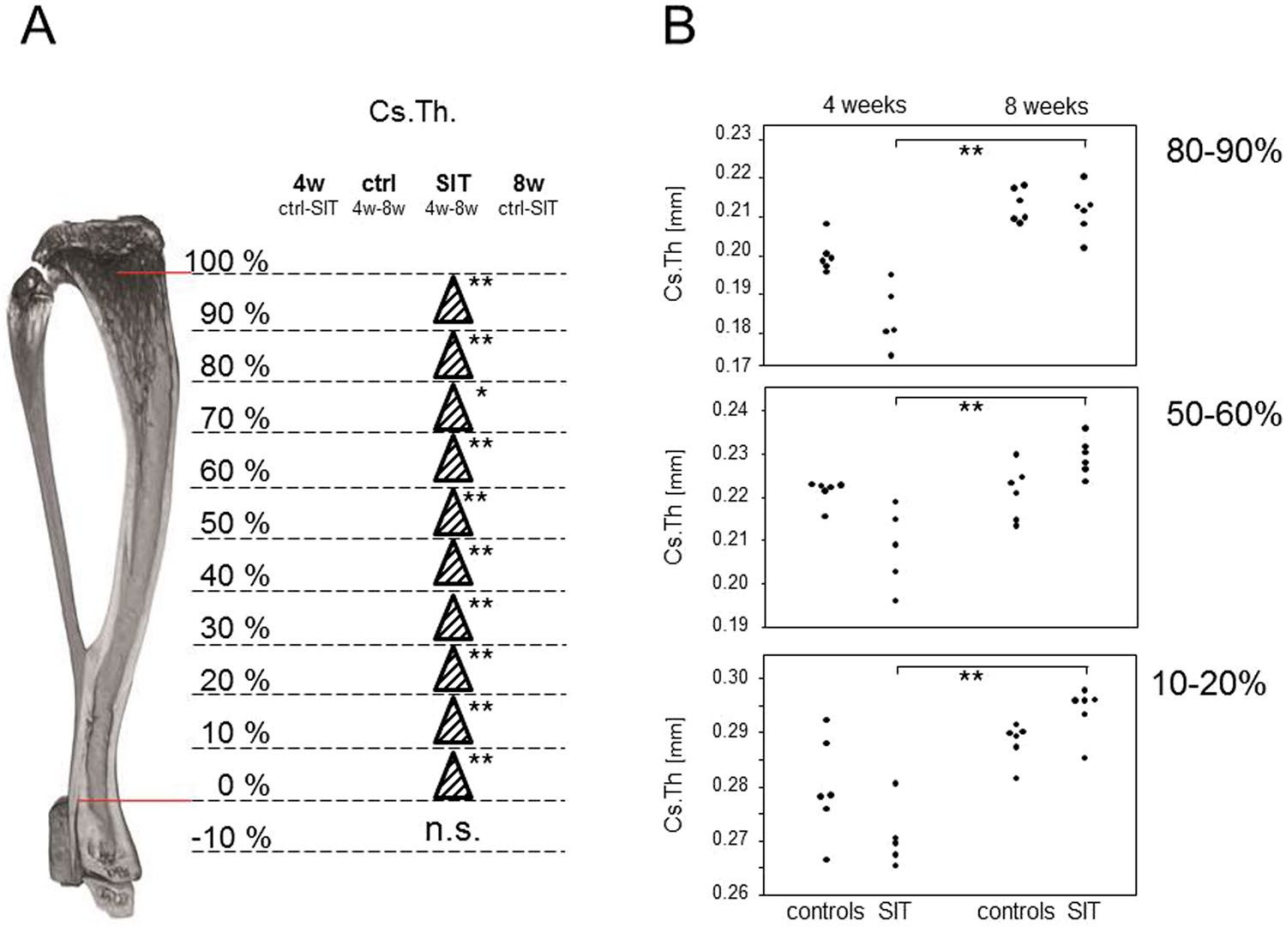
Sprint interval training related changes to the cortical bone of female tibiae occurred at both, endosteal and periosteal surfaces. (**A**) significant increases in Cs.Th. resulting from Kruskal-Wallis with post tests are marked by triangles. (**B**) The various comparisons made are exemplified by dot plots presenting the Cs.Th. assessed at the proximal metaphysis (80–90%), mid-diaphysis (50–60%) and distal metaphysis (10–20%). *P < 0.05; **P < 0.01.

Female tibiae were analysed at the proximal metaphysis for trabecular bone parameters and the mean data presented in Table 2 showed a mere age related reduction in the Tb.N. for the control mice. In contrast, cortical parameters were assessed over the entire length of the tibia and the results suggested that tibial cortical bone was indeed susceptive to the training. Figure 4 summarizes the results obtained for the Cs.Th.: Comparing the Cs.Th. between females that had been trained for four and eight weeks respectively, yielded significant increases over the full length of the tibiae (Fig. 4A). Interestingly, the comparison of Cs.Th. between mice that had been trained for four weeks and their age matched controls indicated a decrease which however, did not reach significance. Likewise, none of the age related increases in Cs.Th. observed for the controls reached significance (Fig. 4B).

In order to find out whether the SIT related changes to the Cs.Th. occured endosteal or periosteal, the B.Ar. and T.Ar. were evaluated. Kruskal-Wallis tests revealed significant training induced increases in both, T.Ar. and B.Ar., predominantly in the distal half of the tibiae. Even though changes to the B.Ar. were more significant than to the T.Ar, increases of Cs.Th. could not unambiguously be attributed to either endosteal or periosteal surfaces and were likely to have occurred at both. Interestingly, increases in the B.Ar./T.Ar. ratio were found in the proximal half (Table 3). These altered ratios were also due to larger increases in the B.Ar. compared to the T.Ar., even though neither B.Ar. nor T.Ar. by themselves yielded significant increases except for the region between 70 and 80%.

**Table 3:** SIT related changes to the female cortical bone are restricted to sites of age related bone growth.

	B. Ar.				T.Ar.				B.Ar./T.Ar.			
	4 weeks	controls	SIT	8 weeks	4 weeks	controls	SIT	8 weeks	4 weeks	controls	SIT	8 weeks
	ctrl-SIT	4w–8w	4w–8w	ctrl-SIT	ctrl-SIT	4w–8w	4w–8w	ctrl-SIT	ctrl-SIT	4w–8w	4w–8w	ctrl-SIT
90–100%												
80–90%											+	
70–80%			+								+	
60–70%											+	
50–60%			++				+				+	
40–50%			++				+					
30–40%			++				+					
20–30%			++				+					
10–20%			++				+					
0–10%			++				+					
−10–0%												

Kruskal-Wallis tests with post tests resulted in significant increases marked by +. Significance levels are denoted by the numbers of symbols: P < 0.5: one symbol; P < 0.1: two symbols.

The results concerning the female hind legs can be summarized as follows: i) only the cortical bone of the tibiae was responsive to the SIT and showed alterations in the Cs.Th., ii) these alterations included a transient and not quite significant decrease after four weeks of SIT, iii) a very significant increase after eight weeks of SIT with iii) changes occurred at both, endosteal and periosteal surfaces with an emphasis on the distal half of the tibiae.

### Lower limb volume was sex specific but did not increase in response to the sprint interval training

**Figure 5:**
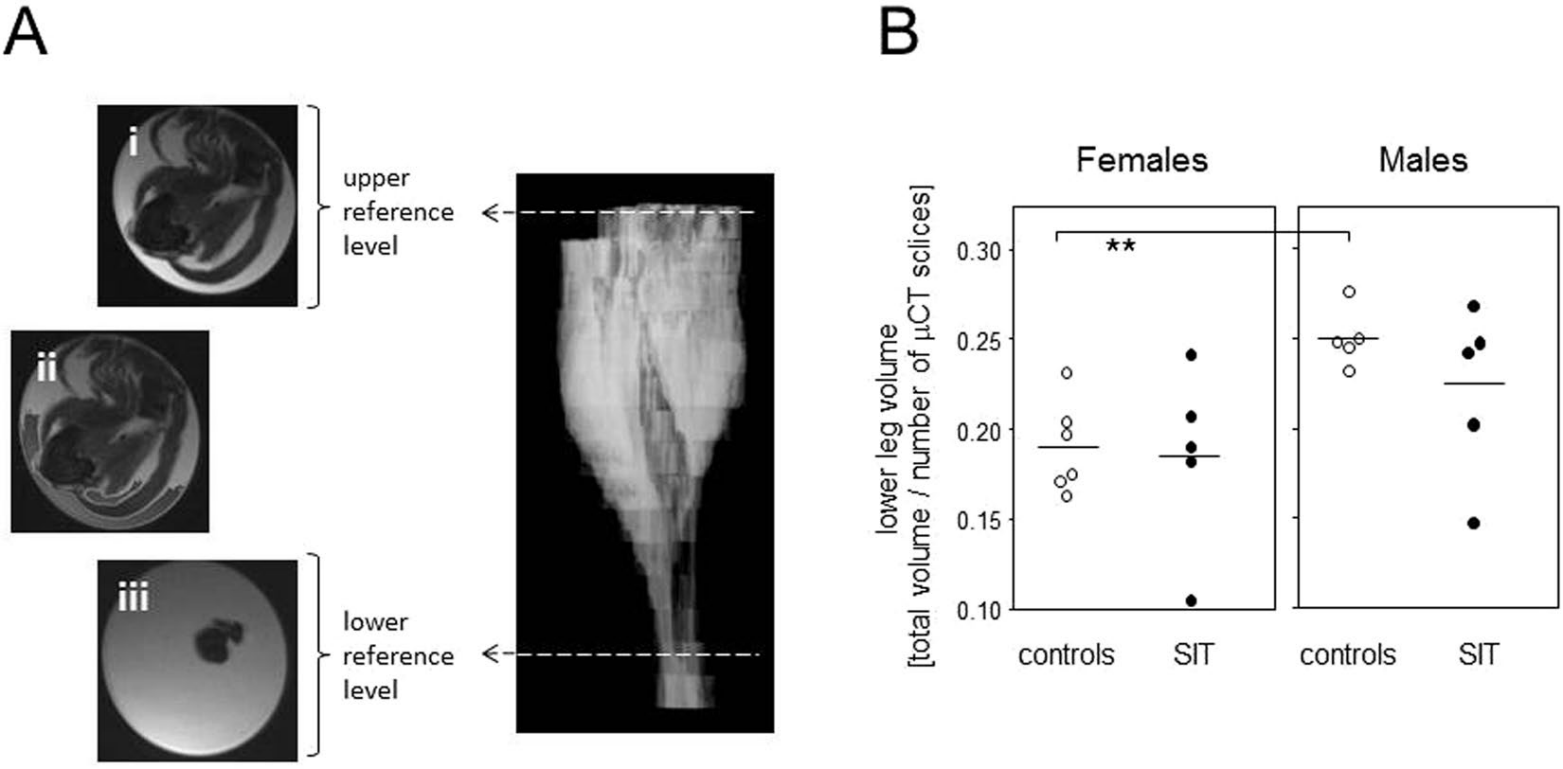
Lower limb volume did not increase in response to the sprint interval training. (**A**) MRI was used to determine the lower hind limb volumes by defining upper (i, tibial plateau) and lower (iii, tibiafibular syndesmosis) reference levels analogous to the µCT analysis, fading out of the regions of no interest (ii) and subsequently relating the lower limb volume to the numbers of µCT slices between upper and lower reference levels. (**B**) The dot plot presents calculated lower leg volumes, each symbol represents a different mouse. Mann-Whitney tests were performed to calculate differences between trained and control mice.

We finally aimed to assess whether the exercise-induced changes observed for the tibiae were paralleled by changes in the muscle volume. As the muscle contributes most to the lower limb volume, we performed a volumetric MRI analysis of the total lower hind limb as a proxy (Fig. 5A). Figure 5B illustrates that there were significant differences between the untrained males and females. However, eight weeks of SIT did not yield any changes in the lower hind limb volumes, neither in the males nor in the females.

## Discussion

The truly innovative aspect of our study is the combination of sprint interval training tailored to the individual performance limits with a detailed µCT analysis that monitored changes to the tibiae over the entire length of the bone. Our results show that male and female cortical bones responded differently to this very strenuous training regimen with the males presenting bone loss and the females presenting bone accrual. Moreover, the male tibiae responded at the distal metaphyses where all loads converge at the smallest cross section. In contrast, female tibiae responded over the entire length of the tibiae. And finally, our cross sectional analyses allowed for the detection of a transient net reduction of female bone mass after four weeks of training and a subsequent increase.

This latter observation is suggestive of two scenarios, either i) SIT in the females induced a true remodelling with the first four weeks of training inducing bone resorption followed by an adaption and formation phase or ii) a detrimental training frequency at five times per week in the beginning that could be compensated for during four weeks of subsequent training at reduced frequency. The loss of male bone at the distal tibia could - in line with this scenario – simply represent a delayed onset of remodelling in male mice.

The present study is a direct continuation of former experiments^8^ and differs from previously described bone stimulating training programs by the use of sprint intervals as opposed to endurance running. The aim behind our sprint interval training was to increase the frequency of the strain and to add a systemic impact that resulted from exceeding the anaerobic threshold. We thus picked up on the hypothesis that an increased bone accrual can be achieved by increasing the frequency of the impact, the intensity of the strain or a combination of both^6,7^. However, our previous study on the high bone mass mouse STR/ort already showed that female and male mice responded differently to the training^8^. While the female STR/ort bones remained unaffected by the SIT, the males showed a diaphyseal decrease of tibia’s cortical bone and a reduced femoral strength. Our present findings now confirm for C57BL/6 mice, that it is again the male cortical bone mass that suffers from the highly strenuous interval training while the females’ cortical bone masses are either unaffected or improve. Of note, the significantly higher weight of the males will result in an even higher strain on the bone, considering that males and females ran at comparable speeds and step frequencies in our experiments. The fact that there was more volume to the male than to the female lower leg and this presumably meant more muscle mass, this may not have sufficed to cushion detrimental loads to the male bone.

We were intrigued by the observation that eight weeks of sprint interval training did not result in an increased volume of the lower legs, even though increased performance limits of the mice confirmed an effect of the training. We cannot exclude however, that more sophisticated methods are needed to differentiate very small changes in muscle volume and future studies should therefore aim at analysing function, e.g. muscle strength or fascicle arrangements and pennate patterns in order to not miss qualitative changes.

Interestingly, Wallace et al^9^. demonstrated that gentle treadmill exercise at 12 m/min, performed daily for three weeks, led to diaphyseal bone accrual in the tibia of male C57BL6/129 mice. Female tibiae in the Wallace experiments did not respond to the training. Of note, the speed of 12 m/min corresponds to the lowest active recovery speed run in our experiments. It therefore seems suggestive that male and female C57BL/6 J mice indeed benefit from different training regimen. Moreover, by confirming that our SIT not only leads to loss of cortical bone in the high bone mass STR/ort but also in the low bone mass C57BL/6 J mice^14^, we were able to show that it is not the underlying bone phenotype that impacts on the bone’s capacity to respond to physical strain but rather the sex^8^. Indeed, previous publications on the role of the bone phenotype on the response to physical activity are conflicting^15,16^. We therefore suggest that in addition to the genes controlling the bone phenotype, factors need to exist that regulate the bones responsiveness to physical strain.

We are aware that our cross sectional analysis only approximates an evaluation of remodelling and does not replace histomorphometry. Indeed, the lack of subsequent histomorphometry at the sites of maximum bone loss or accrual poses a limitation to our study as we can neither determine true remodelling, nor the mineral apposition rate nor can we distinguish between endosteal and periosteal bone accrual nor lamellar and woven bone. Of note, histomorphometry has amply been used in the past^17–19^ however, without a detailed knowledge about the location of maximal bone remodelling, these results may be difficult to interpret. Comparing the findings of multiple research labs is even more difficult, as different animal species, different kinds of sport, different intensities and even different bones were analysed^5^. Future experiments will therefore need to combine µCT and histomorphometry intelligently. Last but not least, the relatively short duration of our study poses a further limitation. The tibial bone loss observed for the males after eight weeks of training could be the beginning of a remodelling process and subsequent bone accrual may emerge in a longer study.

In summary, we here show that sprint interval training tailored to the individual performance limits is suitable to increase cortical bone mass in female C57BL/6 J mice at early adulthood. In contrast, males showed a reduction of cortical bone in the distal tibia. The fact that both, the high bone mass STR/ort and the low bone mass C57BL/6 males showed negative responses to the physical strain disproves our first hypothesis, that the underlying bone mass regulates bone accrual after physical strain. Our second hypothesis, that exercise-induced changes to the bone occur in the distal tibia holds true for males only and is associated with cortical bone loss. In contrast, females not only show accrual of the cortical bone but they do so over the entire length of the tibia. Due to our analysis of trabecular bone at the very centre of tibial and femoral metaphyses, we cannot rule out that trabecular changes occurred in immediate vicinity to the cortical bone. However, early publications already showed that in rodents the trabecular bone is less responsive to treadmill training and this held also true for vertebrae^11,12,20^.

Our results call for follow-up experiments in order to analyse the impact of sprint interval training on the bone of elder and castrated animals and to identify the factors that mediate male bone loss.

## Materials and Methods

### Mice

C57BL/6 J mice were originally purchased from Charles River Laboratories (Wilmington, MA, USA). They were subsequently bred in our central animal care facility under conventional holding conditions, housed in cages with a 12 hour light/dark cycle and given water and food ad libitum. The local state’s animal care committee (Landesamt für Landwirtschaft, Lebensmittelsicherheit und Fischerei M-V; www.lallf.de) approved all experiments (7221.3-1.1-050/13) and all experiments were carried out in accordance with the relevant guidelines and regulations.

### Treadmill training

Treadmill training was voluntary (meaning no punishment in form of e.g. electric shocks) and followed a standardized protocol as described before^8^. In short, mice (24 females, 10 males) were recruited for the treadmill (Process Control Treadmill, TSE Systems, Germany) at the age of six to eight weeks and were then familiarized with the treadmill for two weeks. At the end of these two weeks, the mice performed their first run-to-exhaustion-test (R-T-E) This R-T-E served to identify the individual V_max_, defined as the maximum speed each mouse was capable of running voluntarily. Its results were needed to tailor the training program to the individual performance limits.

#### Run-To-Exhaustion-test

The test followed a ramp up protocol slightly modified from Ingalls *et al*.^21^ after a short warming up at a speed of 10.2 m/min for 3 minutes, the speed was increased over the next 2 minutes to 15 m/min. After another two minutes - and then again every three minutes - the speed was increased in steps of 3 m/min. The test was terminated when the animals no longer kept pace with the treadmill belt and was swept onto the platform behind the running belt.

**Figure 6:**
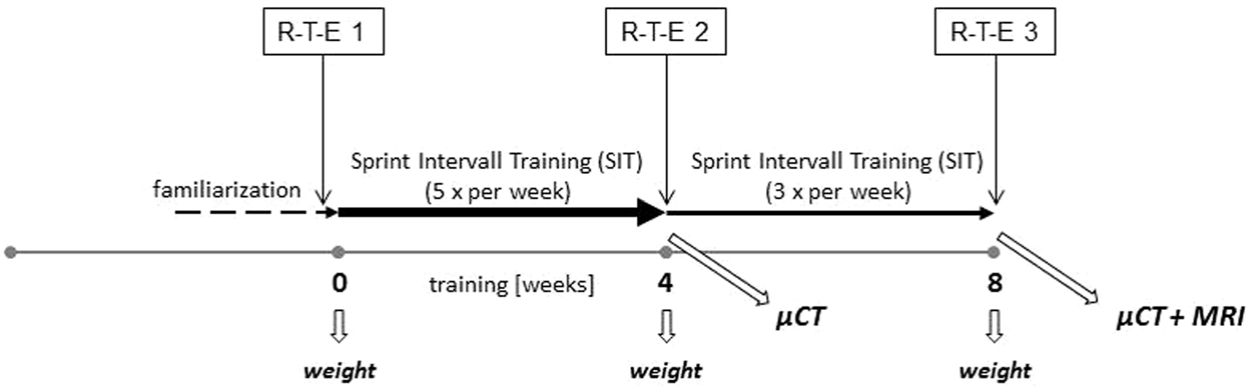
Experimental design. Treadmill training consisted of two weeks of familiarization followed by four weeks of increased frequency sprint interval training (SIT) at five times per week. Thereafter, mice were either sacrificed or went on to another four weeks of SIT at reduced frequency (three times per week). Maximum running speeds were determined via run-to-exhaustion tests (R-T-E) at three time points, before the SIT started (R-T-E 1), after four weeks (R-T-E 2), and again after eight weeks of training (R-T-E 3). Mice were weighed immediately before each R-T-E. Micro CT imaging was performed after the second R-T-E in mice that underwent four weeks of training only and after the third R-T-E in mice that underwent eight weeks of training. MRI imaging was performed on mice that underwent eight weeks of training, only.

The highest speeds achieved represented the individual maximal velocity (Vmax). This test was repeated after four weeks of training, after which mice were either sacrificed or moved on to another four weeks of training which were completed by a third R-T-E (Fig. 6). Each sprint interval training (SIT) lasted 30 minutes and consisted of a warming up period of six minutes followed by four intervals of 1.5-minutes run at peak velocity interspersed by 3.5 minutes of active recovery. The acceleration and deceleration lasted 30 seconds, respectively. Peak velocities aimed at 80% of Vmax, active recovery aimed at 40% of Vmax. For practical reasons, treadmill training was performed in groups of six mice maximum and 100% Vmax corresponded to the mean of the individual Vmax of all mice in a given group. Sprint interval training during the first four weeks consisted of five, the subsequent four weeks of training consisted of three training sessions per week. Allocation to running and control groups was at random. There was no drop out because an animal refused to run at all.

### X-ray micro-computed tomography (μCT)

**Figure 7:**
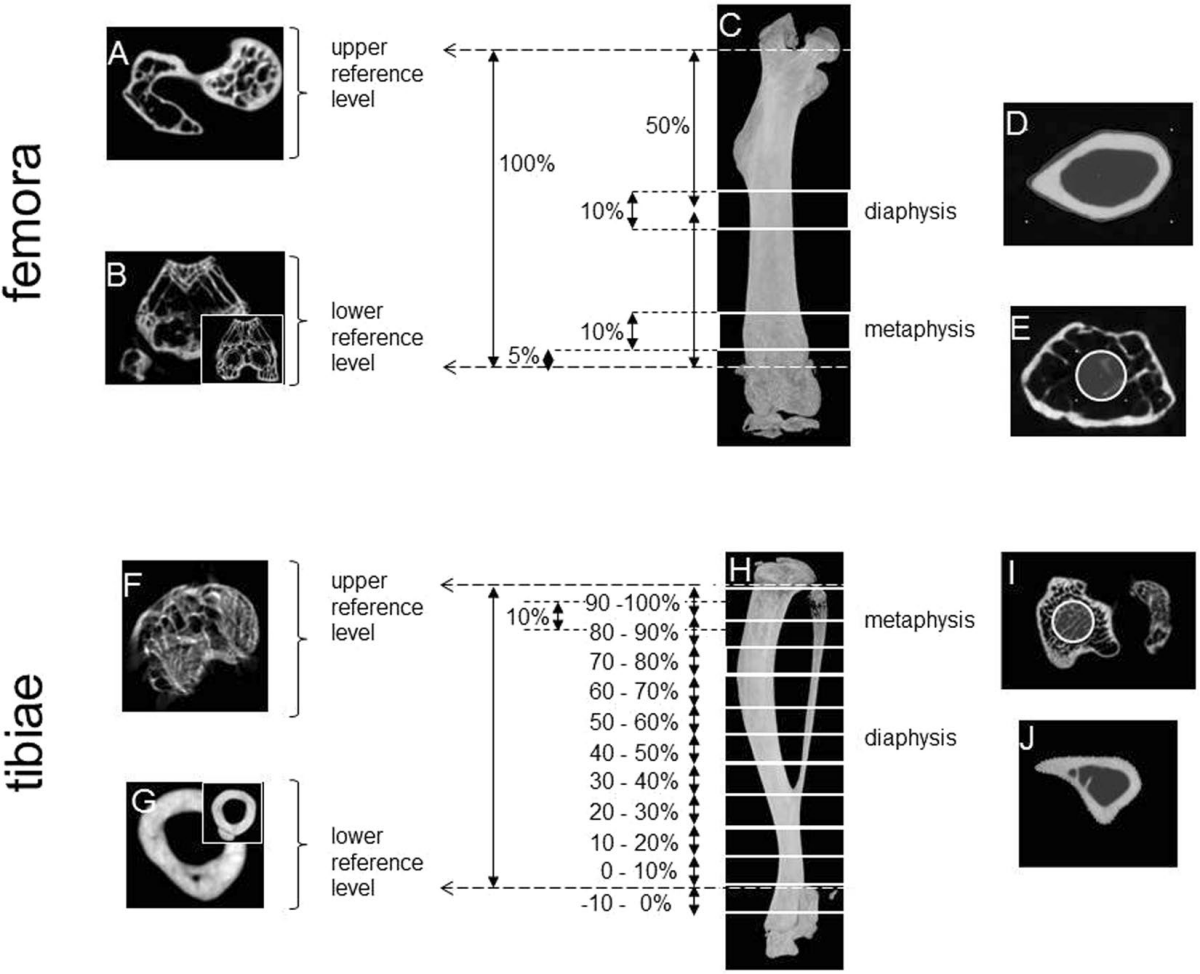
Reference levels and regions of interest for µCT analysis. For the femora (**C**), the fusion of greater trochanter and femoral head (**A**) was defined as upper reference level and the transition from low density cartilage to bone primary spongiosa (insert in **B**) at the distal metaphyseal growth plate (**B**) was defined as lower reference level. For the tibiae (**H**), the proximal epiphyseal growth plate (**F**) was defined as upper and the most distal end of the tibia-fibular syndesmosis (**G**) was defined as lower reference levels. For better orientation, the inserts in **B** and **G** are shown as areas below the respective reference level. The regions of interest (ROIs) for cortical bone (**D**,**J**) correspond to the outline of the respective bone at mid-diaphysis, the ROIs for trabecular bone (**E**,**I**) correspond to circles with diameters half the length of the maximum cross section at metaphyses.

Hind legs were prepared as previously described with the exception that only the skin was removed while the muscle remained intact for further MRI analysis^8^. Computer tomography image acquisition of the hind legs was done by a Bruker SkyScan 1076 (Antwerp, Belgium, SN = 09H02066, Software Version 4.2, 0.5 mm Al-filter, isotropic voxel size 9 µm at 49 kV and 200 µA, rotation step of 0,5°, averaging frame of 3). Image processing was done using NRecon (Micro Photonics), Data viewer (Bruker) and CTAnalyser (Bruker) software. The µCT images were first processed by NRecon software using a Gaussian filter with a smoothing kernel of 2, a defect pixel masking of less than 20%, a ring artefact reduction of 6 and a beam hardening correction of 30%. Afterwards the bones, either femora or tibiae, were vertically orientated and rotated in the same way by using Data viewer software. Finally, image analysis was done by CTAnalyser software using a global threshold of 64–255 and manufacturers 2D and 3D algorithms for the analyses of cortical and trabecular bone, respectively. Bone mineral density (BMD) was determined by using two calcium hydroxide apatite BMD calibration rods with densities of 0.25 g/cm³ and 0.75 g/cm³ for calibration. The regions of interest for image analyses were chosen as follows (see also Fig. 7): The femoral reference levels were defined between the fusion of greater trochanter and the femoral head (upper reference level) and the distal metaphyseal growth plate (GP) where the low density cartilage meets bone primary spongiosa. The tibial reference levels were set between the proximal epiphyseal growth plate (upper reference level) and the tibia-fibular syndesmosis (lower reference level).

10% of the femoral length at mid-diaphysis were analysed for their cortical bone area fractions (Bone Area/Tissue Area = B.Ar./T.Ar.) and cross-sectional thicknesses (Cs.Th.). For the tibia, the cortical bone was analysed in more detail by defining ten intervals of 10% each within the upper and lower reference level plus one of identical size below the lower reference level.

Trabecular bone analyses within the femoral metaphyses started 5% above the lower reference level and comprised 10% of the total femoral length. For the tibial metaphyses, trabecular bone analyses started 5% below the upper reference level and again comprised 10% of the total tibial length. Trabecular bone phenotypes were assessed by analyzing the bone volume fraction (Bone Volume/Tissue Volume = BV/TV), trabecular thickness (Tb.Th.), trabecular number (Tb.N.) and structure model index (SMI). Regions of interest (ROIs) for trabecular bone analysis were defined as circles in the centre of each cross-sectional image. The diameters of these circles corresponded to 50% of the outer femoral and tibial diameters, respectively. Of note, even though we may have missed trabecular changes in immediate vicinity of the cortical boundary, we can definitively rule out any overlay of trabecular parameters by cortical ones.

### Magnetic resonance imaging (MRI)

MRI was used to determine the total volume of the lower hind leg. Even though this total volume comprised not only the bone but also all soft tissues, we anticipated that changes in muscle volume would outshine alterations in all other tissues. As the lower limb volume also depends on length, the ratio of total volume to length (calculated as the numbers of µCT image slices between lower and upper reference levels) was used for statistical analysis.

MRI was performed on a 7 Tesla Bruker BioSpec 70/30 (gradient system: BGA 12 S HP, 86 mm transmit volume coil, 2 × 2 receive surface coil, Bruker Biospin, Ettlingen, Germany) using a T2 weighted TurboRARE (Rapid Acquisition with Relaxation Enhancement) sequence with the following settings: TR: 7457.3 ms, TE: 36.7 ms, field of view: 23.8 × 12.8, matrix size: 235 × 128, in-plane resolution 100 × 100 µm, slice thickness: 0.6 mm, 65 slices, no slice gap, RARE-factor: 8. The MR images were analyzed using the Aquarius iNtuition software (TeraRecon, Foster City, California, USA, ver. 4.4.12.185.3539). Lower and upper reference levels were aligned to the µCT data (Fig. 7H–J). After fading out the surrounding ethanol by use of the software’s “threshold tool” (Fig. 5A ii), an automated calculation of the remaining total volume was performed.

### Statistics

Statistical analyses were performed using either GraphPad (GraphPad Software, CA, USA), IBM SPSS Statistics 22 (IBM, NY, USA) or SigmaPlot 13.0 (Systat Software, CA, USA). Data were tested for normality using the Shapiro-Wilk test. Comparisons between groups (N smaller or equal 6) were performed via Mann–Whitney U test (Figs 1, 2A, 5B). Kruskal-Wallis with post tests were performed for multiple comparisons (Figs 3 and 4, Tables 1, 2 and 3). Correlation coefficients for data following Gaussian distribution were calculated according to Pearson (Fig. 2B). P values lower than 0.05 were considered significant.
